# Nanopowder Metal Oxide for Photoluminescent Gas Sensing

**DOI:** 10.1186/s11671-017-1891-5

**Published:** 2017-02-20

**Authors:** V. M. Zhyrovetsky, D. I. Popovych, S. S. Savka, A. S. Serednytski

**Affiliations:** 1 Pidstryhach Institute for Applied Problems of Mechanics and Mathematics NASU, Naukova Str., 3b, Lviv, 79060 Ukraine; 2 National University “Lvivska Polytechnika”, Bandera Str., 12, Lviv, 79013 Ukraine

**Keywords:** Nanopowder, Gas sensor, Metal oxide, Photoluminescence

## Abstract

Gas sensing properties of metal oxide nanopowders (ZnO, TiO_2_, WO_3_, SnO_2_) with average diameters of 40–60 nm were analyzed by room-temperature photoluminescence spectroscopy. The influence of gas environment (O_2_, N_2_, H_2_, CO, CO_2_) on the emission intensity was investigated for metal oxide nanopowders with surface doped by impurities (Pt, Ag, Au, Sn, Ni or Cu). Established physicochemical regularities of modification of surface electronic states of initial and doped nanopowders during gas adsorption. The nature of metal oxide nanopowder gas-sensing properties (adsorption capacity, sensitivity, selectivity) has been established and the design and optimal materials for the construction of the multi-component sensing matrix have been selected.

## Background

Sensors for active, in particular, toxic and explosive gases play an important role in the monitoring and control of the environment, especially in highly industrialized regions. Gas sensors based on nanostructured metal oxides have shown great potential for the detection of different gases, in particular, toxic, hazardous, or inflammable gases such as H_2_, CO, NO_2_ or NH_3_ [1–3]. Such sensors can also be applied in medicine, particularly for diagnosis of cancer [4]. Nanopowders have excellent sensing properties due to their high surface-to-volume ratio [5, 6], which has high adsorption ability and reactivity [7]. Among semiconductor sensors, metal oxide nanopowders are of interest as nontoxic, easy-to-handle, and highly sensitive materials that can be grown with excellent crystal quality using different physical and chemical fabrication techniques [8–12]. In addition, the use of different cover metal layers (Pt, Ag, Au, Sn, Ni or Cu) as catalysts for gas chemisorption or physisorption can achieve an increase in the detection limit for specific gases and could be in the range of parts per billion (ppb) [13–15].

Gas sensing is performed mostly by electrical measurement. The adsorption of oxidative and reductive gases on the surface of nanopowders leads to a change in the charge density on the surface and a shift of the Fermi level. This induces a change in the electron distribution in the nanomaterial [16–18]. A wide range of adsorption centers leads to low selectivity of material, and consequently we need to find ways of improving this. The difficulty of selective detection is due to in particular to a similar mechanism of interaction of the reductant gases with the surface of metal oxides. The sensitivity of metal oxides to the nature and concentration of adsorbed molecules largely depends on the surface microstructure, which can be modified both by laser annealing and by the formation of complex heterogeneous systems, including the metal-semiconductor type “core-shell” [19]. The solution to this problem can also be to use arrays of metal oxide nanopowders covered with different functional additives that affect the adsorption process [20].

Several publications on luminescent gas sensing are available [18–25]. An advantage of luminescent sensing is that it avoids electrical contact between the nanostructures and the resulting metallic impurities. Also, real-time information about the change of specific contributions in the photoluminescence spectra can be observed. However, the direct effect of adsorbed gases on the photoluminescence of metal oxide nanopowders has not yet been adequately studied.

This work aims at solving the problem of increasing the selectivity of the gas-sensitive materials and accordingly, gas sensors in general. Its feature is an integrated approach to solving problems that is to use the methods of fabrication of nanopowders and structures on the basis that we have developed [19, 21, 26] and to apply the photoluminescent method for the detection of adsorbed gas particles on the surface of metal oxide nanopowders [20, 21]. This approach is promising due to its very high inherent sensitivity and miniaturization capability.

## Methods

The research into phase and structural analyses of nanopowders was carried out using an x-ray diffractometer, DRON-4. The sizes of particles were determined according to electron microscopy images received by means of the electron microscope, PEM-125 K.

Doping of nanopowders was conducted by pulsed laser deposition of thin films on the nanopowders with further activation by laser annealing [27, 28]. Pulsed evaporation of target material was carried out using a neodymium-doped yttrium aluminium garnet (Nd:YAG) laser (λ = 1,06 μm, τ = 10^-3^–10^-8^ s, q = 10^5^÷10^8^ W/cm^2^, n = 14–56 Hz, d = 5 mm, E_i_ = 0.005–0.350 J). Laser radiation was focused on the target located in a vacuum chamber (P = 10^-3^ Pa), so we obtained chemically pure condensates of activator. The plasma plume was directed perpendicular to the nanopowder level in the cuvette. Dopant atoms condensed on the surface of the powder and formed the thin film of dopant. The cuvette containing the nanopowder was placed on the vibration device (oscillation frequency 10–60 Hz); this provided an even film of condensation over the entire surface of the nanopowder. The vibrating oscillations provide good mixing of powder, allowing even deposition of film on its surface. With the purpose of activating the deposited impurities and its diffusely uniform distribution in the volume of granule was carried out laser annealing of nanopowder. Under the action of pulsed laser emission the alloying metallic impurities from the surface diffuses into granules and is electrically activated. Laser heating and implantation carried out in the transparency band of nanopowders and therefore with the absorption of radiation on the metallic impurity and without significant heating of nanopowders.

Photoluminescent research into gas environments were conducted using the computerized device with the dual monochromator DMR-4. Photoluminescence excitation was performed using UV light-emitting diodes (LEDs) with λ_max_ = 355 or λ_max_ = 375 nm. The photoluminescent study was carried out at room temperature. The investigated samples were placed in a quartz cuvette connected to the vacuum installation VUP-5 M and the multi-system gas inlet SNA-2, which made it possible to conduct photoluminescent research in various gas environments at a given range of pressures. Registration of the signal was carried out using the photomultiplier FEU-27. Recording and normalization of spectra was conducted automatically using specially developed software.

## Results and Discussion

The metal oxide nanopowder samples were obtained by means of pulse laser-reactive technology [17, 19]. X-ray diffraction studies show that ZnO samples have a wurtzite crystal structure and an average diameter of nanoparticles of 40–60 nm.

**Fig. 1:**
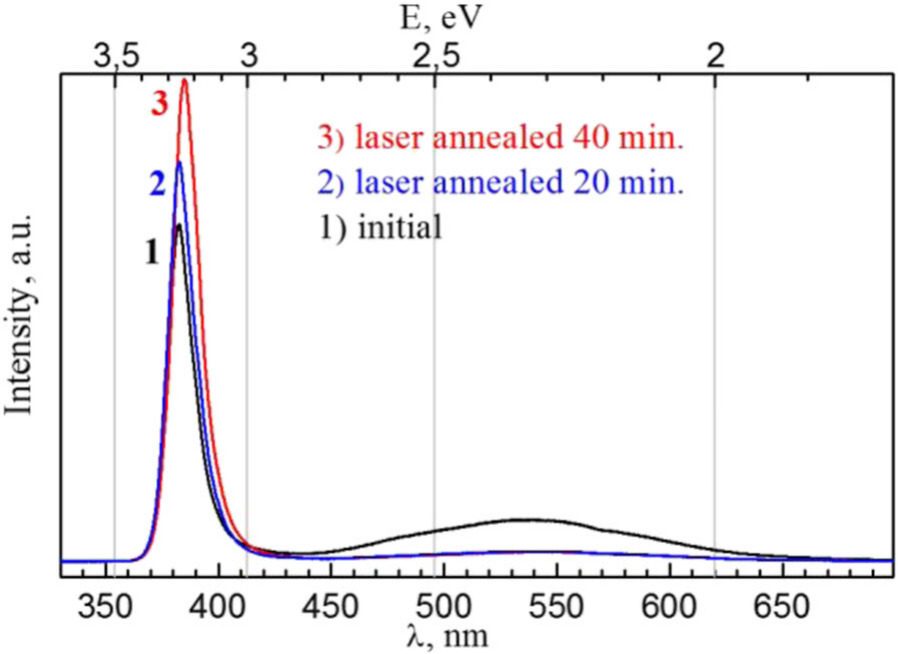
Photoluminescence spectra of initial (1) and laser annealed at 20 minutes (2) and 40 minutes (3) ZnO nanopowders

The photoluminescence spectra measured at room temperature for initial and laser-annealed ZnO nanopowders (technological settings to obtain E_i_ = 4,9 J/cm^2^, τ_i_ = 120 ns, P_O2_/P_He_ = 1/3) are shown in Fig. 1. All obtained spectra consist of two major emission bands the intensive UV (λ_max_ = 385 nm) and visible band at around 540 nm. The intensive UV peak corresponds to the exciton luminescence of ZnO at room temperature. The intensity of edge luminescence is stronger than the intensity of visible luminescence from the intrinsic defect structure, indicating the excellent crystal quality of ZnO nanoparticles and underdevelopment of the intrinsic defect structure, which can be modified by means of pulsed laser annealing. The character of visible photoluminescence determined by the intrinsic defect structure of the material and depends on the technological parameters obtaining of nanopowders [26].

**Fig. 2:**
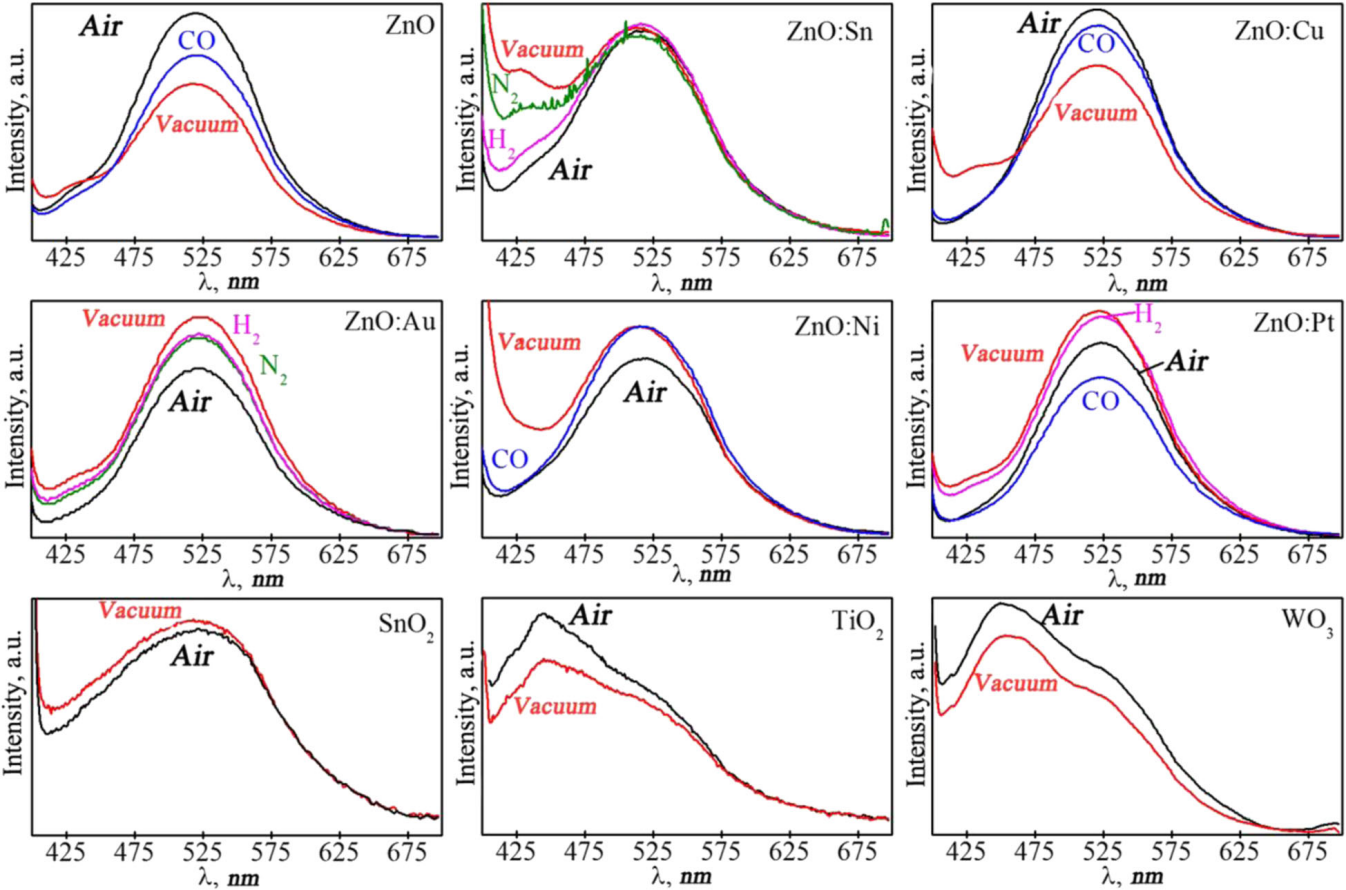
Photoluminescence spectra of metal oxid nanopowders (ZnO, TiO_2_ SnO_2_, WO_3_) in different gas environments

Figure 2 shows the photoluminescence spectra of metal oxide nanopowders (ZnO, ZnO:Sn, ZnO:Cu, ZnO:Ni, ZnO:Pt, ZnO:Au, TiO_2,_ SnO_2_, WO_3_) in various gas environments. The use of catalysts deposited on the surface of the material as a finely dispersed phase not only leads to an increase in the set selectivity, but also to an increase in the sensitivity of the sensor in relation to the selected gas. This is brought about by the directed change in the condition of surface and creation of active centers of selective chemical interaction for detecting gas. There is a simultaneous decrease in the amount of non-selective interaction centers, much of which is associated with oxygen vacancies. The mechanism of influence of the deposited catalysts on the value and characteristics of the adsorption response of the sensor and its selectivity are often associated with the spillover effect [14, 29], which is linked to change in the Fermi level of the semiconductor adsorbent.

The influence of the deposited catalysts on the sensitivity and selectivity of the semiconductor sensors can also be shown through adsorption and change in the characteristics of the space charge region, which is located directly under contact with the catalytic metallic impurity – the semiconductor, caused by adsorption. The processes that occur during adsorption on metal impurities cause changes in the electronic subsystem and changes in the characteristics of the Schottky barrier, which is demonstrated by changes in the concentration of free carriers in the space charge region that participate in the luminescent process.

**Fig. 3:**
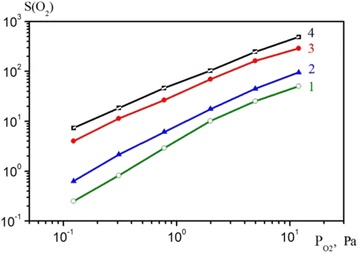
Sensor sensitivity of the nanopowders ZnO (*1*), ZnO:Ag (*2*), ZnO:Au (*3*) and ZnO:Pt (*4*), dependent on the additional partial pressure of oxygen in the air

The sensor sensitivity of the ZnO nanoparticles before and after the deposition of catalysts is shown in Fig. 3. To understand the effect of the platinum coating on sensitivity of ZnO nanoparticles to oxygen, we first must consider the mechanism of the sensitivity of uncoated nanoparticles. It is known that the depletion layer plays a key role in the mechanism of sensitivity. Formation of the depletion layer takes place in accordance with the available oxygen vacancies in ZnO, which act as traps to capture oxygen molecules. Usually, increasing the thickness of the depletion layer increases the resistance of ZnO. One of the ways to increase the sensitivity of ZnO nanoparticles to reducing gases such as CO, is by increasing the number of trapped electrons from adsorbed oxygen, so obtaining a larger depletion layer, and therefore the maximum change in luminescence.

The observed increase in the sensitivity of ZnO nanoparticles to oxygen is probably caused by the combination of two effects, namely electronic sensitization and as a result of this, the effect of platinum on the surface of metal oxide ZnO. Sensitization is due to differences in the work function between regions of covered platinum and uncovered ZnO nanoparticles. In comparison with ZnO, platinum has a higher value of work function. This means that the Fermi level of ZnO nanoparticles is lower than for platinum, which leads to the transfer of some free electrons from ZnO nanoparticles to the platinum cover, as both systems achieve thermodynamic equilibrium, changing the position of the Fermi level.

Reducing the number of free electrons in ZnO nanoparticles causes an increase in the thickness of the depletion layer [14]. The spillover effect causes increased dissociation of oxygen molecules to oxygen ions. Thus, a weak coupling is formed between the oxygen molecule and the platinum atom. This weak coupling can be easily broken with the formation of oxygen ions that diffuse to the surface vacancies of ZnO nanoparticles. Thus, there are more trapped electrons, which increase the sensitivity of ZnO nanoparticles to gases. The mechanism of the catalytic ability of gold on the surface of ZnO nanoparticles can be twofold. On the surface of a gold cluster there is active interaction of oxygen with gold and additional activation of chemisorbed oxygen on the border of the three-phase system, Au-ZnO-O_2_.

The sensor system predicted by us is based on the registration photoluminescence spectra of metal oxide nanopowder with adsorbed gas particles [20]. The energy levels of electrons are created in the adsorbent by adsorbed particles, which allow us to observe individual electron spectral levels of the adsorbed atoms and selectively identify them.

**Fig. 4:**
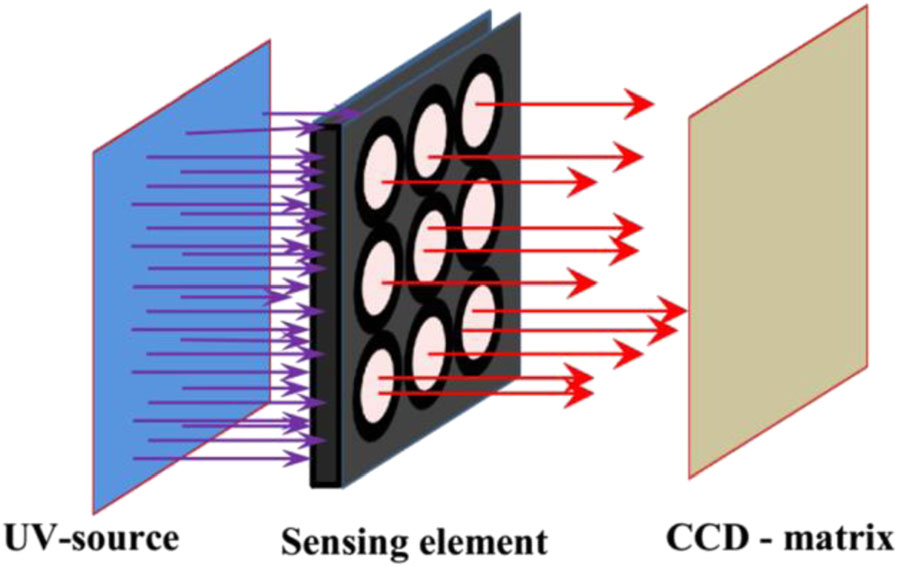
Schematic diagram of the system with a multi-component sensing matrix and a charge coupled device (CCD)

**Fig. 5:**
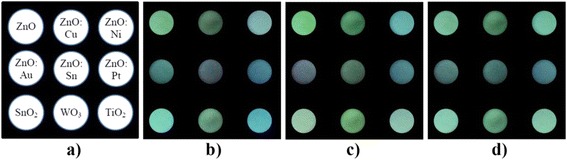
Photoluminescent emissions cells of the multi-component sensing matrix (**a**) in different gas environments: air (**b**), CO (**c**) and vacuum (**d**)

For practical application of the proposed sensor and to increase its selectivity, we propose to use a multichannel sensor system (Fig. 4), which has a set of metal oxide adsorbents of various modifications, characterized by different sensitivity to various gas particles. Simultaneous measurement signals from all matrix cells (Fig. 5) by a charge-coupled device (CCD) and digital processing provide an opportunity to increase selectivity analysis and to at the same time to determine the concentration and type of many active gases adsorbed on the surface of metal oxide.

## Conclusions

The luminescent properties of metal oxide nanopowders (ZnO, TiO_2_, SnO_2_, WO_3_) have been studied in various gas environments (O_2_, N_2_, H_2_, CO, CO_2_) for use in gas sensing. The effect on gas sensing properties (adsorption capacity, sensitivity and selectivity) of a surface doped by impurities (Pt, Ag, Au, Sn, Ni or Cu) was investigated. We proposed a multichannel luminescent sensor system, which has a set of metal oxide adsorbents of various modifications, which are characterized by different sensitivity to various gas particles. Simultaneous measurements of the luminescent signals of cells in this system provide an opportunity to determine the qualitative and quantitative composition of the components in the gas environment.
